# A clinical‐radiomic‐pathomic model for prognosis prediction in patients with hepatocellular carcinoma after radical resection

**DOI:** 10.1002/cam4.7374

**Published:** 2024-06-12

**Authors:** Qu Xie, Zeyin Zhao, Yanzhen Yang, Xiaohong Wang, Wei Wu, Haitao Jiang, Weiyuan Hao, Ruizi Peng, Cong Luo

**Affiliations:** ^1^ Department of Hepato‐Pancreato‐Biliary & Gastric Medical Oncology Zhejiang Cancer Hospital, Hangzhou Institute of Medicine (HIM), Chinese Academy of Sciences Hangzhou Zhejiang China; ^2^ Wenzhou Medical University Wenzhou Zhejiang China; ^3^ Molecular Science and Biomedicine Laboratory (MBL), State Key Laboratory of Chemo/Biosensing and Chemometrics, College of Chemistry and Chemical Engineering, College of Biology, Aptamer Engineering Center of Hunan Province, Hunan University Changsha Hunan China; ^4^ Zhejiang Cancer Hospital, Hangzhou Institute of Medicine (HIM), Chinese Academy of Sciences Hangzhou Zhejiang China; ^5^ Department of Intestinal Oncology Zhejiang Cancer Hospital, Hangzhou Institute of Medicine (HIM), Chinese Academy of Sciences Hangzhou Zhejiang China; ^6^ Department of Pathology Zhejiang Cancer Hospital, Hangzhou Institute of Medicine (HIM), Chinese Academy of Sciences Hangzhou Zhejiang China; ^7^ Department of Radiology Zhejiang Cancer Hospital, Hangzhou Institute of Medicine (HIM), Chinese Academy of Sciences Hangzhou Zhejiang China; ^8^ Department of Intervention Zhejiang Cancer Hospital, Hangzhou Institute of Medicine (HIM), Chinese Academy of Sciences Hangzhou Zhejiang China

**Keywords:** hepatocellular carcinoma, machine learning, pathomics, radiomics, recurrence

## Abstract

**Purpose:**

Radical surgery, the first‐line treatment for patients with hepatocellular cancer (HCC), faces the dilemma of high early recurrence rates and the inability to predict effectively. We aim to develop and validate a multimodal model combining clinical, radiomics, and pathomics features to predict the risk of early recurrence.

**Materials and Methods:**

We recruited HCC patients who underwent radical surgery and collected their preoperative clinical information, enhanced computed tomography (CT) images, and whole slide images (WSI) of hematoxylin and eosin (H & E) stained biopsy sections. After feature screening analysis, independent clinical, radiomics, and pathomics features closely associated with early recurrence were identified. Next, we built 16 models using four combination data composed of three type features, four machine learning algorithms, and 5‐fold cross‐validation to assess the performance and predictive power of the comparative models.

**Results:**

Between January 2016 and December 2020, we recruited 107 HCC patients, of whom 45.8% (49/107) experienced early recurrence. After analysis, we identified two clinical features, two radiomics features, and three pathomics features associated with early recurrence. Multimodal machine learning models showed better predictive performance than bimodal models. Moreover, the SVM algorithm showed the best prediction results among the multimodal models. The average area under the curve (AUC), accuracy (ACC), sensitivity, and specificity were 0.863, 0.784, 0.731, and 0.826, respectively. Finally, we constructed a comprehensive nomogram using clinical features, a radiomics score and a pathomics score to provide a reference for predicting the risk of early recurrence.

**Conclusions:**

The multimodal models can be used as a primary tool for oncologists to predict the risk of early recurrence after radical HCC surgery, which will help optimize and personalize treatment strategies.

## 1 INTRODUCTION

Hepatocellular carcinoma (HCC) is a prevalent cancer globally, ranking fifth in incidence and third in cancer‐related deaths.^1^ Radical surgery remains the primary method for curing HCC due to strict transplant conditions and a limited supply of liver donors for transplantation.^2^ However, HCC has a high recurrence rate post‐surgery, with rates ≥10% annually and reaching 30%–50% after 2 years.^3^, ^4^ Thus, identifying high‐risk factors for postoperative recurrence of HCC and establishing a stable and effective predictive model is of utmost importance for the treatment and prognosis of patients.

As an essential component of standard management for patients with HCC, traditional imaging assessment relies heavily on qualitative features and lacks the ability to identify tumor heterogeneity.^5^, ^6^ Despite improvements in imaging technology, challenges persist in accurately assessing and monitoring tumors.^7^ In recent years, radiomics has been widely used for predicting tumor treatment efficacy, recurrence, and survival through quantitative analysis of high‐throughput images, transforming the qualitative and subjective assessment of conventional imaging into quantifiable and reproducible standardized image information.^8^, ^9^, ^10^ Studies have demonstrated the potential value of radiomics in high‐throughput quantitative analysis of HCC images to effectively assist in the personalized management of high‐risk patients for tumor recurrence and to characterize and screen patients at high risk of recurrence.^11^, ^12^, ^13^ Although radiomics has shown promising results in predicting tumor recurrence, it is limited by potential hazards, such as overfitting and limited applicability. Therefore, further research is needed to explore and validate the reliability and validity of radiomics.

The wealth of information on pathological features in postoperative tissues provides essential information for clinical diagnosis, treatment, and prognosis.^14^, ^15^ However, the variability in evaluation between pathologists and the semi‐quantitative nature of the scoring system makes it difficult to avoid the problems of reproducibility and poor subjectivity.^16^, ^17^ With the development of digital pathology, the foundation has been laid for rapid identification and accurate quantification of pathological features in pathomics.^18^ Recent studies have shown that pathomics and radiomics can effectively predict tumor recurrence, metastasis risk, and survival, providing an essential reference basis and guidance for clinical treatment and management.^19^ It should be noted that pathomics is different from radiomics in that the former provides deeper microscopic information about tumor cells and subcellular structures. The latter reflects the intensity distribution and spatial relationship of tumor tissues. Therefore, combining pathomics and radiomics can comprehensively capture tumors' macroscopic and microscopic structural features.^20^, ^21^ In recent studies, prognostic models that combine radiomics and pathomics features have demonstrated excellent predictive performance in various cancers, such as colorectal and glioblastoma multiforme.^20^, ^22^ However, in previous studies, explorations on predicting the risk of postoperative recurrence in patients with HCC have mainly focused on predictions based on preoperative computed tomography (CT)/ magnetic resonance imaging (MRI) radiomics and combined clinical features.^23^, ^24^ No studies have used radiomics and pathomics features to predict the risk of postoperative recurrence in patients with HCC.

Therefore, this study aimed to develop and validate a multimodal model using preoperative clinical, radiomics, and pathomics features to predict the risk of postoperative early recurrence in patients with HCC.

## 2 METHODS

### 2.1 Study population

**FIGURE 1:**
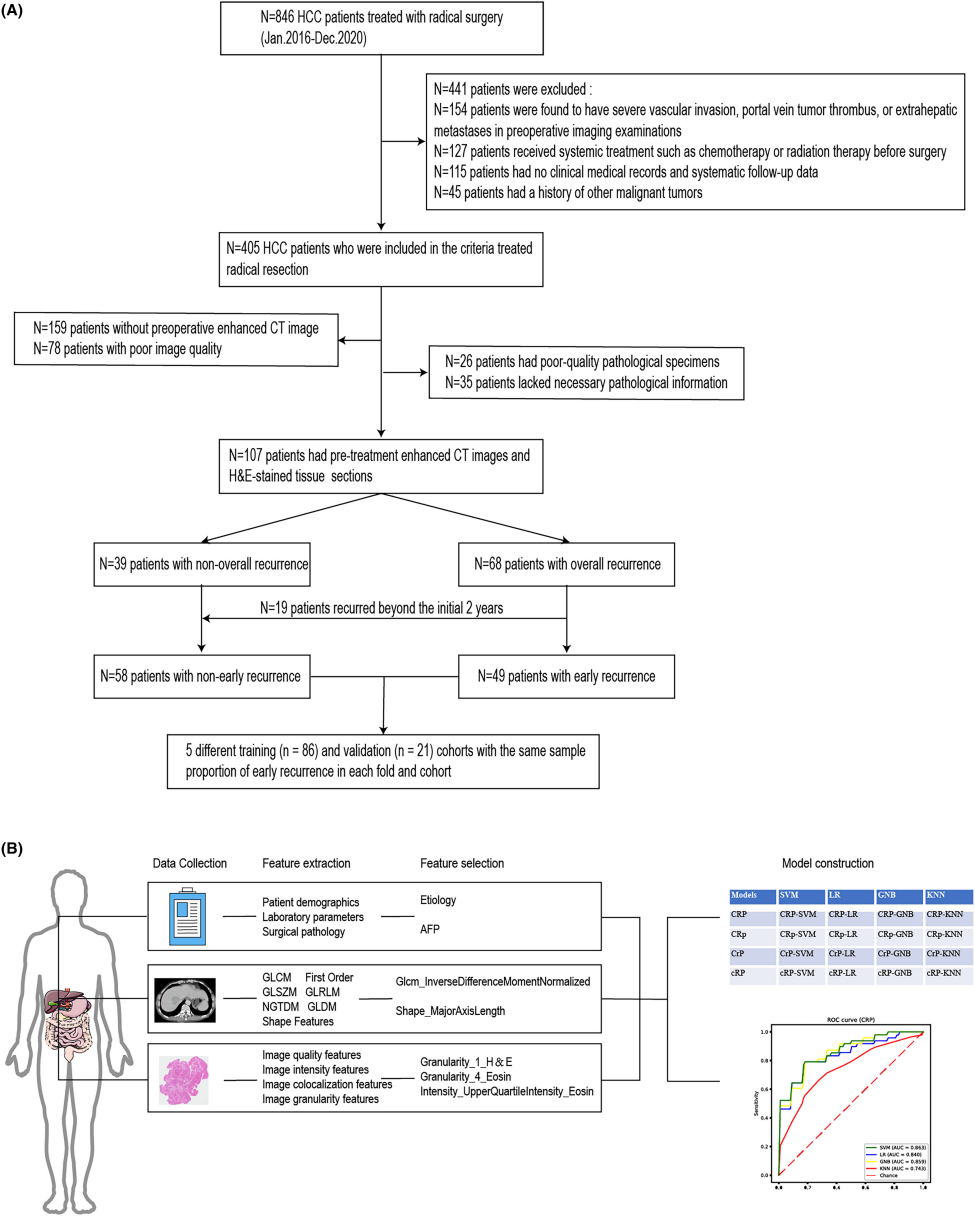
Study population and technical route. AFP, alpha‐fetoprotein; CC, Hepatocellular carcinoma; CrP, Clinical‐Pathomic model; CRp, Clinical‐Radiomic model; CRP, Clinical‐Radiomic‐Pathomic model; cRP, Radiomic‐Pathomic model; enhanced CT, enhanced computed tomography; GLCM, gray level co‐occurrence matrix; GLDM, gray level dependence matrix; GLRLM, gray level run length matrix; GLSZM, gray‐level size zone matrix; GNB, Gaussian Naive Bayes; H & E, hematoxylin and eosin; KNN, K neighbor algorithm; LR, logistic regression; NGTDM, neighboring gray tone difference matrix; ROC curve, receiver operating characteristic curve; SVM, Support Vector Machine.

This study aimed to develop a multimodal clinical‐radiomic‐pathomic model to predict postoperative early recurrence risk in patients with HCC who underwent radical resection. To achieve this goal, we conducted a retrospective analysis of data from patients who underwent radical resection for HCC at Zhejiang Cancer Hospital between January 2016 and December 2020 (Figure 1A).

The inclusion criteria for the study were as follows:

1. Patients who did not receive neoadjuvant therapy such as chemotherapy, radiotherapy, or interventional treatment;
2. Patients who underwent enhanced CT examination within 1 month before surgery;
3. Complete clinical history and standardized and systematic follow‐up information at least 6 months of follow‐up;

The exclusion criteria were as follows:

1. Patients with a previous history of other malignancies;
2. Patients with severe vascular invasion, portal vein aneurysm emboli, or extrahepatic metastases identified on preoperative imaging;
3. Patients who died within 30 days after surgery due to surgical complications;

The study was conducted following the principles of the Declaration of Helsinki. The Ethics Committee of Zhejiang Cancer Hospital approved this retrospective study, and the requirement for informed patient consent was waived (IRB‐2022‐503).

### 2.2 Clinical characteristics

The preoperative assessment included blood tests, cardiac function assessment, chest and abdomen CT scans, and pulmonary function tests. The resectability of the lesion was judged based on the results of the preoperative assessment, and the staging was determined according to the Barcelona Clinic Liver Cancer (BCLC) staging system.^25^ A team of expert liver cancer surgeons was responsible for performing all the surgeries.

The relevant clinical data included (a) demographic and clinical characteristics, such as age, gender, etiology, liver cirrhosis, BCLC staging and adjuvant therapy; (b) laboratory variables, including total protein (TP), albumin (ALB), albumin to globulin ratio (AGR), aspartate aminotransferase (AST), alanine aminotransferase (ALT), alkaline phosphatase (ALP), gamma‐glutamyl transpeptidase (GGT), total bilirubin (TBIL), direct bilirubin (DBIL), c‐reactive protein (CRP), white blood cell (WBC) count, neutrophil (Neut) count, lymphocyte (Lymp) count, neutrophil to lymphocyte ratio (NLR), platelet (Plt) count, platelet to lymphocyte ratio (PLR), international normalized ratio (INR), prothrombin time (PT), alpha‐fetoprotein (AFP), carcinoembryonic antigen (CEA) and Child–Pugh class; (c) histopathological features, including tumor size, number, capsule invasion, and nerve invasion.

### 2.3 Follow‐up

After surgery, patients were followed up every 3 months in the first 3 years, every 6 months from the third to fifth year, and annually after that. The monitoring plan included physical examinations, serum AFP, and enhanced CT or MRI of the chest and abdomen. The endpoint of this study was recurrence‐free survival (RFS), defined as the time interval from the date of surgery to the first recurrence, metastasis, or the date of the last follow‐up. Based on the RFS interval, we defined whether recurrence occurred within 2 years after surgery as early recurrence and whether recurrence occurred up to the last follow‐up as overall recurrence (Figure 1A, Table 1, and Table S1). In addition, overall survival (OS) was defined as the interval between surgery and death or the date of the last follow‐up.

**TABLE 1:** Baseline characteristics of early recurrence.

Characteristic	Without early recurrence (*n* = 58)	Early recurrence (*n* = 49)	Univariable logistic regression		Multivariable logistic regression	
			OR (95%CI)	*p*	OR (95%CI)	*p*
Patient demographics						
Age (mean [SD])	58.81 (9.67)	56.84 (11.37)	0.982 (0.946,1.019)	0.332		
Sex (female/male)	7 (12.1)/51 (87.9)	7 (14.3)/42 (85.7)	0.824 (0.268,2.535)	0.735		
Etiology (HBV/Other)	42 (72.4)/16 (27.6)	44 (89.8)/5 (10.2)	0.298 (0.100,0.887)	**0.030**	0.131 (0.029,0.593)	**0.008**
Liver cirrhosis (NO/Yes)	23 (39.7)/35 (60.3)	24 (49.0)/25 (51.0)	0.685 (0.317,1.476)	0.334		
BCLC staging (0orA/B)	55 (94.8)/ 3 (5.2)	36 (73.5)/13 (26.5)	6.620 (1.762,24.878)	**0.005**	4.287 (0.582,31.570)	0.153
Adjuvant therapy (No/Yes)	38 (65.5)/20 (34.5)	24 (49.0)/25 (51.0)	1.979 (0.908,4.313)	**0.086**	1.387 (0.513,3.747)	0.519
Laboratory parameters						
TP (≤65/>65 g/L)	7 (12.1)/51 (87.9)	9 (18.4)/40 (81.6)	0.610 (0.209,1.780)	0.366		
ALB (≤40/>40 g/L)	16 (27.6)/42 (72.4)	18 (36.7)/31 (63.3)	0.656 (0.290,1.486)	0.312		
A/G (≤1.5/>1.5 g/L)	9 (15.5)/49 (84.5)	8 (16.3)/41 (83.7)	0.941 (0.333,2.660)	0.909		
ALT (≤50/>50 U/L)	42 (72.4)/16 (27.6)	38 (77.6)/11 (22.4)	0.760 (0.314,1.840)	0.543		
AST (≤40/>40 U/L)	39 (67.2)/19 (32.8)	27 (55.1)/22 (44.9)	1.673 (0.762,3.670)	0.200		
ALP (≤45/>45 U/L)	53 (91.4)/5 (8.6)	43 (87.8)/6 (12.2)	1.479 (0.422,5.179)	0.540		
GGT (≤60/>60 U/L)	35 (60.3)/23 (39.7)	22 (44.9)/27 (55.1)	1.868 (0.864,4.036)	0.112		
TBIL (≤20/>20 μmol/L)	52 (89.7)/6 (10.3)	44 (89.8)/5 (10.2)	0.985 (0.281,3.447)	0.981		
DBIL (≤7/>7 μmol/L)	52 (89.7)/6 (10.3)	45 (91.8)/4 (8.2)	0.770 (0.204,2.903)	0.700		
CRP (≤10/>10 mg/L)	51 (87.9)/7 (12.1)	40 (81.6)/9 (18.4)	1.639 (0.562,4.784)	0.366		
WBC (≤3.5/>3.5 × 10^9^/L)	2 (3.4)/56 (96.6)	3 (6.1)/46 (93.9)	0.548 (0.088,3.418)	0.519		
Neut (≤1.8/>1.8 × 10^9^/L)	1 (1.7)/57 (98.3)	4 (8.2)/45 (91.8)	0.197 (0.021,1.828)	0.153		
Lymp (≤1.1/>1.1 × 10^9^/L)	11 (19.0)/47 (81.0)	4 (8.2)/45 (91.8)	2.633 (0.781,8.876)	0.118		
NLR (≤3/>3)	46 (79.3)/12 (20.7)	35 (71.4)/14 (28.6)	1.533 (0.631,3.725)	0.345		
Plt (≤125/>125 × 10^9^/L)	10 (17.2)/48 (82.8)	10 (20.4)/39 (79.6)	0.812 (0.307,2.150)	0.676		
PLR (≤125>/125)	43 (74.1)/15 (25.9)	41 (83.7)/8 (16.3)	0.559 (0.214,1.459)	0.235		
INR (≤1/>1)	56 (96.6)/2 (3.4)	48 (98.0)/1 (2.0)	0.583 (0.051,6.634)	0.664		
PT (≤14/>14 seconds)	53 (91.4)/5 (8.6)	48 (98.0)/1 (2 0.0)	0.221 (0.025,1.958)	0.175		
AFP (≤400/>400 ng/mL)	31 (53.4)/27 (46.6)	8 (16.3)/41 (83.7)	5.884 (2.353,14.715)	**<0.001**	6.421 (2.163,19.055)	**0.001**
CEA (≤5/>5 ng/mL)	53 (91.4)/5 (8.6)	44 (89.8)/5 (10.2)	1.205 (0.327,4.431)	0.779		
Child–Pugh Class (A/B)	47 (81.0)/11 (19.0)	31 (63.3)/18 (36.7)	2.481 (1.033,5.961)	**0.042**	1.534 (0.490,4.798)	0.462
Surgical pathology information						
Tumor number (≤3/>3)	56 (96.6)/2 (3.4)	42 (85.7)/7 (14.3)	4.667 (0.922,23.619)	**0.063**	1.269 (0.095,17.019)	0.857
Tumor size (≤5/>5 cm)	43 (74.1)/15 (25.9)	21 (42.9)/28 (57.1)	3.822 (1.690,8.642)	**0.001**	2.199 (0.748,6.468)	0.152
Capsular invasion (No/Yes)	43 (74.1)/15 (25.9)	26 (53.1)/23 (46.9)	2.536 (1.125,5.715)	**0.025**	2.328 (0.774,7.000)	0.133
Neural invasion (No/Yes)	47 (81.0)/11 (19.0)	34 (69.4)/15 (30.6)	1.885 (0.771,4.611)	0.165		

*Note*: The bold values are used to highlight statistically significant values for our study.

Abbreviations: AGR, albumin to globulin ratio; ALB, albumin; ALP, alkaline phosphatase; ALT, alanine aminotransferase; AST, aspartate aminotransferase; BCLC staging, Barcelona Clinic Liver Cancer; CEA, carcinoembryonic antigen; CRP, c‐reactive protein; DBIL, direct bilirubin; GGT, gamma‐glutamyl transpeptidase; HBV, Hepatitis B virus; INR, International Normalized Ratio; Lymp, lymphocyte; Neut, neutrophil; NLR, neutrophil to lymphocyte ratio; PLR, platelet to lymphocyte ratio; Plt, platelet; PT, prothrombin time; TBIL, total bilirubin; TP, total protein; WBC, white blood cell.

### 2.4 Extraction of radiomics features from enhanced CT images

All patients underwent preoperative enhanced CT scanning of the abdomen, which included the acquisition of arterial‐phase and portal‐phase images. For radiomic analysis, we focused on the portal venous phase CT images. This choice was based on the advantages of portal‐phase images in clearly delineating the boundaries of HCC tumors, as well as their ability to highlight the unique blood supply features of the tumor. These features are essential for the accurate extraction of radiomic features that reflect tumor biological behavior and treatment response. Therefore, we selected portal vein stage CT images from the image archiving and communication system (PACS) of Zhejiang Cancer Hospital and exported them to 3D Slicer open‐source software (www.slicer.org/) for accurate image segmentation. A radiologist manually outlined the region of interest (ROI) of the primary lesion in 3D at each successive level along the contour of the lesion, and a senior radiologist reviewed it. The segmented ROI of the primary lesion was then exported and stored as a Nearly Raw Raster Data (NRRD) image.

Quantitative radiomics features of the ROI were extracted using the PyRadiomics platform (https://pyradiomics.readthedocs.io/en/latest/).^26^ The extracted radiomics features include shape features, first‐order features, gray level co‐occurrence matrix (GLCM), gray‐level size zone matrix, gray level run length matrix, neighboring gray tone difference matrix, gray level dependence matrix. Ultimately, a total of 107 features were extracted from each ROI image of the primary lesion (Table S2).

### 2.5 Extraction of pathomics features from Whole Slide Images (WSI)

Pathology sections are digitally scanned at high resolution using WSI scanning technology at an image magnification of ×20. The scanned images were stored in Virtual Slide Image (VSI) file format to ensure the integrity and accessibility of the image data. A senior pathologist with more than 20 years of experience in liver pathology was responsible for image quality control, and slides with poor staining quality or obvious artifacts were strictly screened out. On this basis, this pathologist further selected five representative and nonoverlapping representative blocks with a field of view of 1000 × 1000 pixels and saved them in tiff format. These plots were then independently reviewed by another senior pathologist. In the event of disagreement between the two pathologists, a third pathologist would be invited to review them to ensure the accuracy and consistency of the assessment.

The open‐source tools of the CellProfiler platform (version 4.2.5, https://cellprofiler.org/) were used to extract the quantitative pathomorphological features of the selected sections.^27^ Firstly, H & E was converted into a gray‐scale image using the “ColorToGray” module, based on the “Combine” method. Then, the color channels of the ROI image were separated into hematoxylin‐stained and eosin‐stained gray‐scale images using the “UnmixColors” module. Subsequently, greyscale H & E, H & E images were assessed using the “MeasureImageQuality” module with three types of features: blur, intensity, and threshold. Next, the “MeasureColocalization” module measured the colocalization and correlation between intensities in hematoxylin images and eosin images on a pixel‐by‐pixel basis. Finally, the “MeasureGranularity” module outpute spectra of size measurements of the textures in three types of images. The summary of the 90 pathohistological features is presented in Table S3.

### 2.6 Feature filtering and calculation of the radiomics score and pathomics score

To reduce overfitting or bias in the model, two feature selection methods, logistic regression and the least absolute shrinkage and selection operator (LASSO), were used to select features on the entire dataset based on the early recurrence subgroups, thus enhancing the model's generalization ability.

Features with *p*‐values less than 0.1 in univariate logistic regression were included in multivariate logistic regression to identify independent predictors of clinical features (Table 1). In contrast, the following steps were performed to screen 107 radiomics and 90 pathomics features, respectively. Firstly, the features were normalized by *z*‐score transformation, thus avoiding the effect of variability between features. Second, Spearman's correlation coefficient was calculated to determine the correlation between features, and one of the features with a correlation coefficient greater than 0.9 between any two features was retained to eliminate redundant information. Further, a Mann–Whitney *U* test was performed on the remaining features to retain significant features. Ultimately, we applied LASSO combined with cross‐validation to select features for potentially relevant risk factors while ensuring best‐fit error.^28^

Radiomics and pathomics scores are based on the entire dataset and calculated by weighting selected Radiomics, Pathomics features and respective coefficients.

## 3 MODEL CONSTRUCTION

### 3.1 Model definition and building

We use four feature types and adopt four algorithms: Support Vector Machine (SVM) (model parameters: kernel type, regularization parameter (C), gamma), logistic regression (LR) (model parameters: regularization parameter, regularization type), Gaussian Naive Bayes (GNB) (model parameters: prior probability, smoothed variance), and K neighbor algorithm (KNN) (model parameters: nearest neighbors number, weights type), to establish 16 models, aiming to compare the best machine learning model for predicting the early recurrence of HCC.^29^, ^30^, ^31^, ^32^ The first model, CRP, includes clinical, radiomics, and pathomics features. The second model, CRp, includes clinical and radiomics features. The third model, CrP, includes clinical and pathomics group features. The fourth model, cRP, includes radiomics and pathomics features.

### 3.2 Five‐fold cross‐validation

In this study, we used a five‐fold cross‐validation approach to reduce over‐fitting. We split the original dataset into five sub‐datasets, four of which are used to train the model and the remaining one to test the model. This process is repeated five times, using one of the sub‐datasets as the test set each time. The final evaluation score is calculated by averaging the scores obtained in the five iterations.^33^

### 3.3 Assessment of model performance

To evaluate the predictive ability of the model in terms of postoperative recurrence, we utilized receiver operating characteristic (ROC) curves. The area under the curve (AUC) was calculated to quantify the model's performance. Furthermore, we employed the confusion matrix to determine the accuracy (ACC), sensitivity, and specificity to assess the diagnostic performance of the model.^34^

### 3.4 Statistical analysis

To assess the differences in clinical variables between early recurrence and nonearly recurrence groups, we employed the Wilcoxon rank sum test for continuous variables and the Chi‐square test or Fisher exact test for categorical variables. Furthermore, univariate and multivariate logistic regression analyses were conducted to determine independent predictors for early and overall recurrence. The Kaplan–Meier method was employed to analyze survival curves, and comparisons were made using the Log‐rank test. Statistical analyses were performed using GraphPad Prism 9.5, and statistical significance was set at *p* < 0.05.

The LASSO analysis and nomogram were generated using the Rstudio. ROC curves were constructed for multiple models to evaluate their prediction accuracy. AUC, ACC, sensitivity, and specificity were utilized to assess the predictive performance of the risk models. Machine learning was conducted using MATLAB 2022b.

## 4 RESULTS

### 4.1 Patient

The study design is shown in Figure 1B. A total of 107 patients met the eligibility criteria between January 2016 and December 2020. The entire cohort's median follow‐up and survival durations were 25.6 and 36.5 months, respectively. Early and overall recurrence occurred in 45.79% (49/107) and 63.55% (68/107) of patients, respectively. Table 1 and Table S1 present the baseline clinical characteristics of patients with early and overall recurrence. Multivariate logistic regression analyses of early and overall recurrence showed that etiology was an independent predictor of HCC after radical resection. In contrast, AFP was an independent predictor of early recurrence.

### 4.2 Feature analysis

**FIGURE 2:**
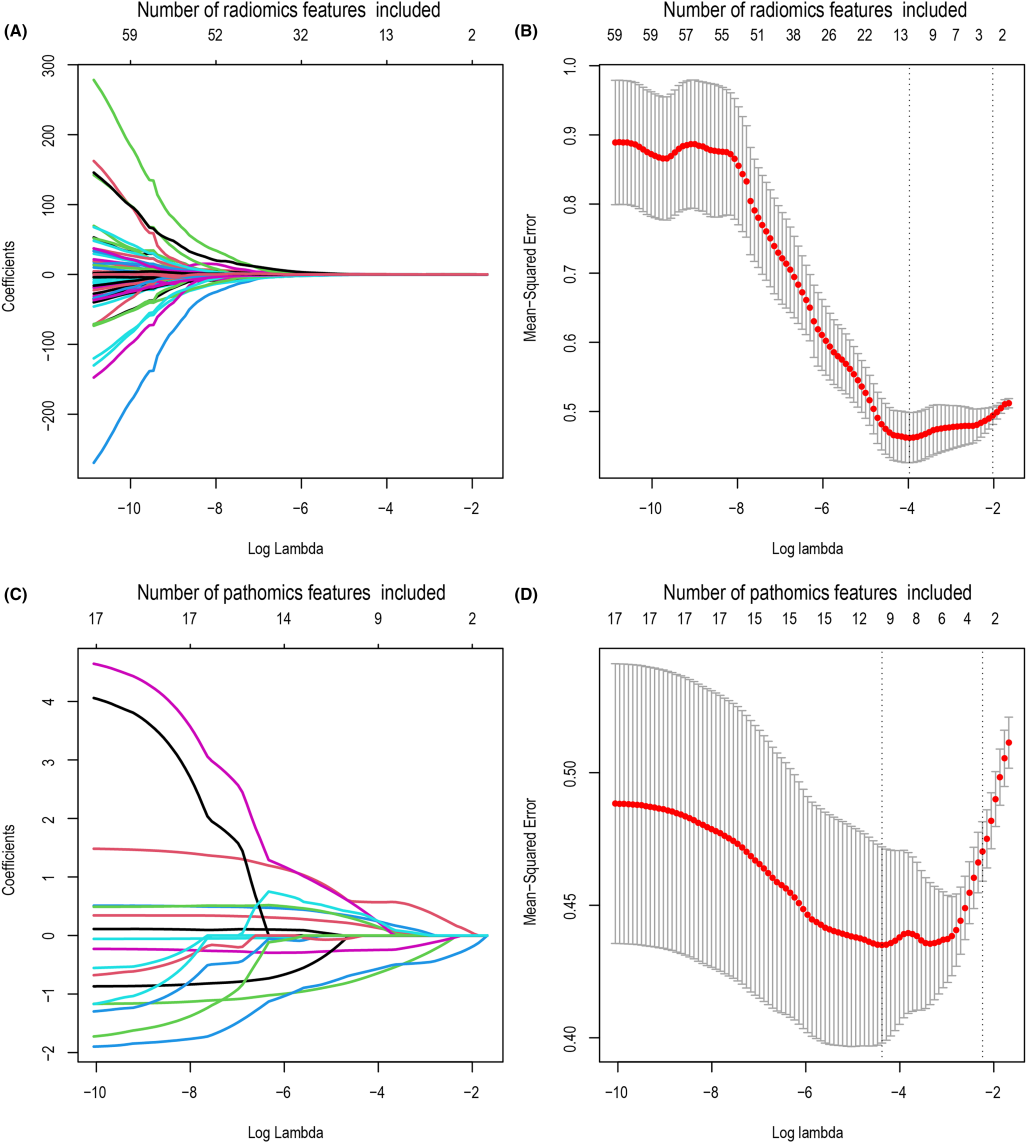
Radiomics and pathomics features selection using the least absolute shrinkage and selection operator (LASSO) regression. LASSO coefficient curves for radiomics (A) and pathomics (C) features. Cross‐validated curves for LASSO regression analysis for radiomics (B) and pathomics (D) parameter lambda selection.

To identify features associated with HCC early recurrence, we conducted LASSO analysis with cross‐validation and found five features, including two radiomics and three pathomics features (Figure 2). We evaluated the robustness of these features by comparing their differences between the early recurrence and without early recurrence groups using the Wilcoxon rank sum test (Figure S1). Ultimately, we arrived at the following expression for the combination of features:

$$\begin{array}{c} \text{Radiomics Score}=0.1165823^{*}\text{Glcm}\_\text{InverseDifferenceMomentNormalized}\\ \;+0.1707390^{*}\text{Shape}\_\text{MajorAxisLength}. \end{array}$$

$$\text{Pathomics Score}=-0.003471338^{*}\text{Granularity}\_1\_H\&E.$$

$$+0.136190427^{*}\text{Granularity}\_4\_\text{Eosin}.$$

$$-0.283946392^{*}\text{Intensity}\_\text{UpperQuartileIntensity}\_\text{Eosin}.$$

### 4.3 Model performance

**FIGURE 3:**
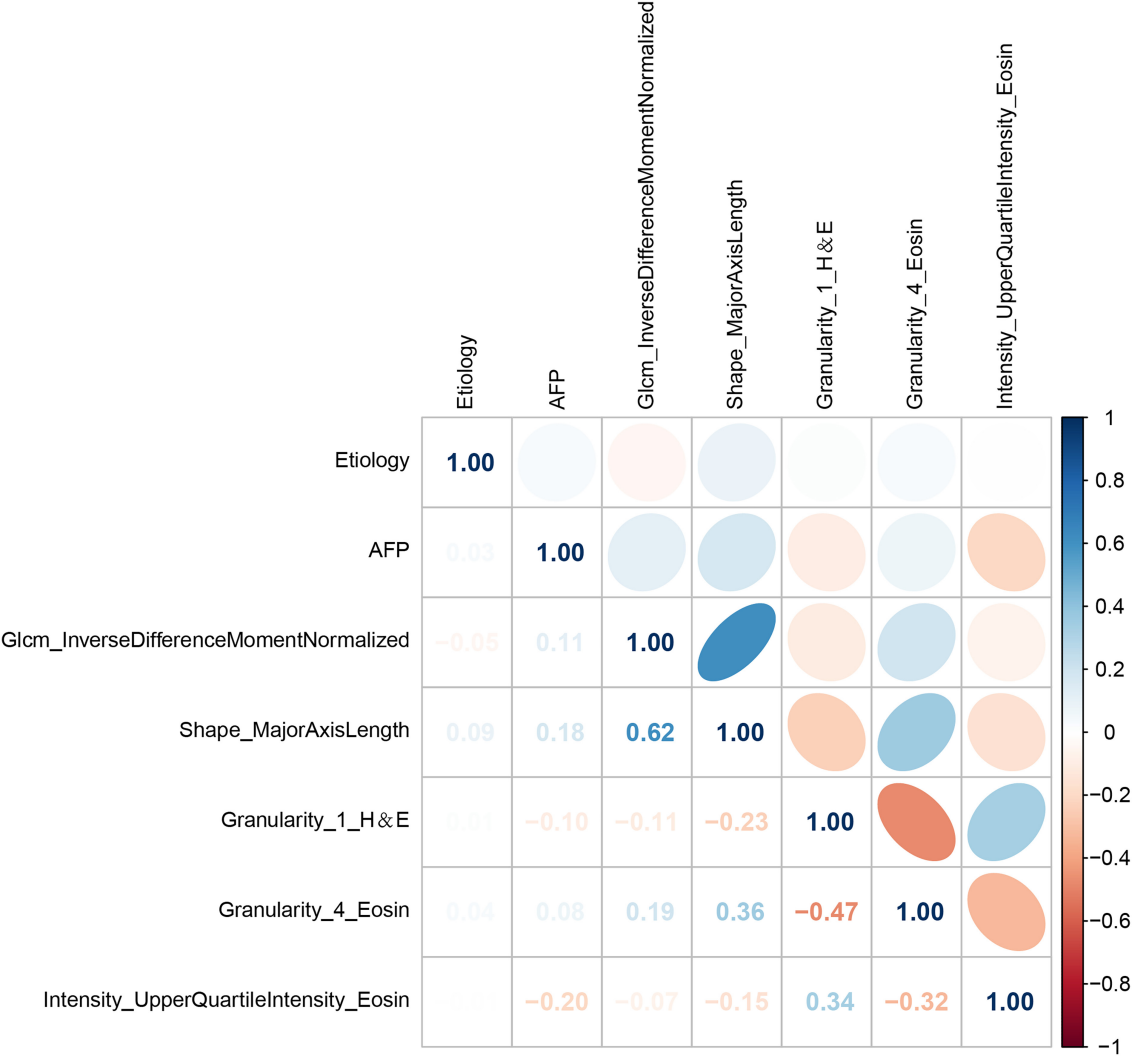
The Heat map between clinical, radiomics and pathomics features.

**FIGURE 4:**
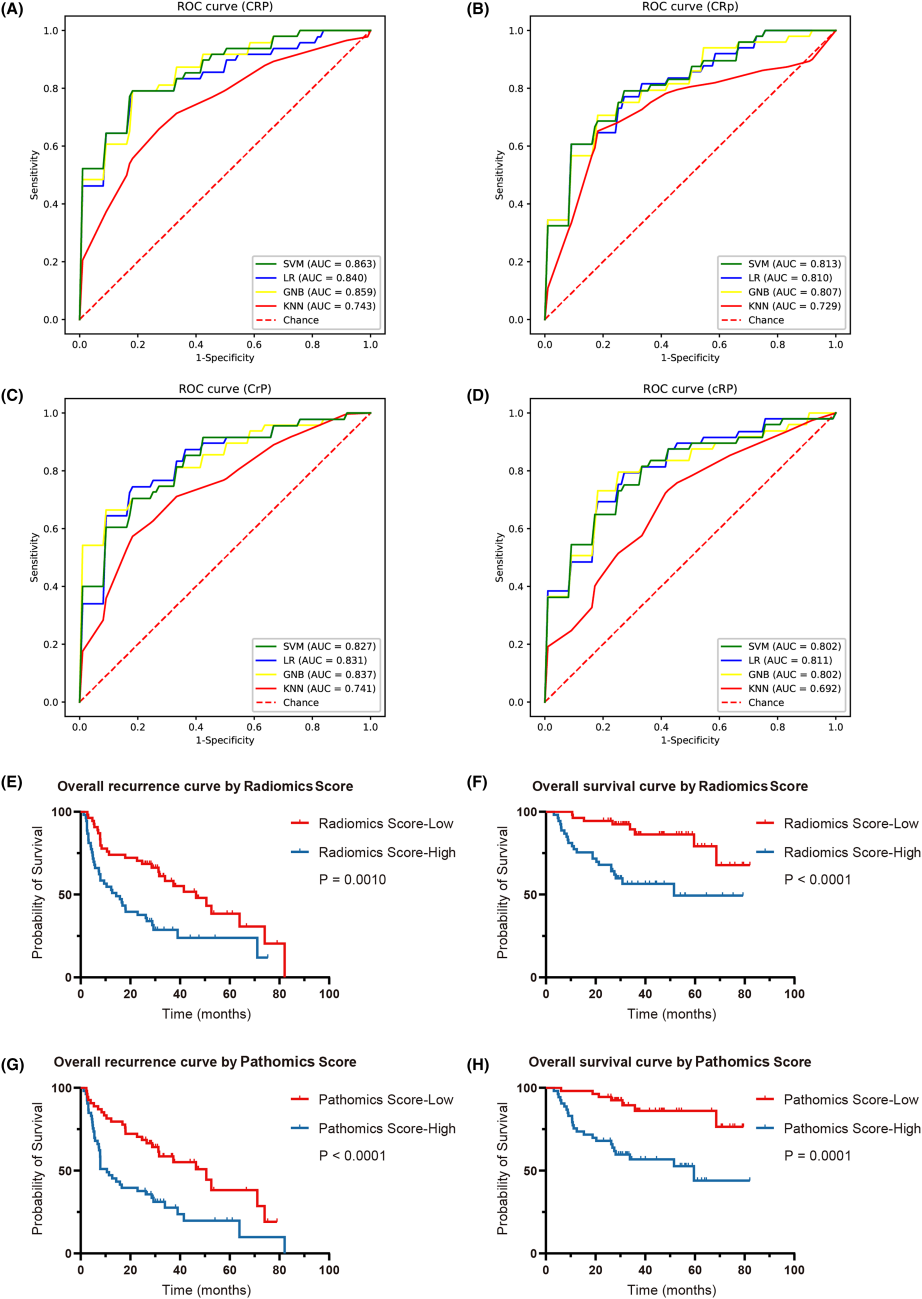
The ROC curves and AUC of early recurrence, and Kaplan–Meier curves for overall recurrence and OS were shown. (A–D) The ROC and AUC curves of different algorithms, models for predicting early recurrence in the validation dataset. (E–G) The proportions based on radiomics score and pathomics score (radiomics or pathomics score ≤ 0.5 and >0.5) were calculated separately using Kaplan–Meier curves for overall recurrence and OS. AUC, area under the curve; ROC curves, receiver operating characteristic curves; OS, Overall survival.

This study utilized nine characteristics associated with early recurrence to develop four prediction models (CRP, CRp, CrP, and rCP). A heat map (Figure 3) presented the degree of association between these seven variables. Evaluation of these models was done using SVM, LR, GNB, and KNN modeling, followed by 5‐fold cross‐model validation and confusion matrix analysis. In addition, Table S4 shows the change in prediction performance over five cross‐validations. The CRP multimodal model outperformed the bimodal models for predicting early postoperative recurrence, with the AUC ranging from 0.743 to 0.863 (Figure 4). The AUC ranged from 0.729 to 0.813, 0.741 to 0.837, and 0.692 to 0.811 for the CRp, CrP, and cRP models, respectively (Figure 4). This suggests that omitting one type of feature set can slightly or moderately impair prediction performance.

Notably, the SVM (best parameters: “kernel type”: “linear,” “regularization parameter (C)”: 1, “gamma”: 1^e‐05^) showed the best performance in the multimodal prediction model (AUC: 0.863, ACC: 0.784, sensitivity: 0.731, specificity: 0.826) (Figure 4, Table 2). In addition, radiomics score and pathomics score effectively stratified HCC outcomes, with higher scores associated with higher overall recurrence risk and OS (Figure 4E–H).

**TABLE 2:** The average AUC, ACC, sensitivity and specificity by 16 models after 5‐Fold Cross‐Validation for Identifying early recurrence.

Models	Classifiers	AUC	ACC	Sensitivity	Specificity
CRP	SVM	0.863	0.784	0.731	0.826
	LR	0.840	0.766	0.731	0.792
	GNB	0.859	0.774	0.691	0.841
	KNN	0.743	0.711	0.651	0.761
CRp	SVM	0.813	0.756	0.731	0.774
	LR	0.810	0.710	0.751	0.671
	GNB	0.807	0.691	0.669	0.705
	KNN	0.729	0.719	0.691	0.741
CrP	SVM	0.827	0.719	0.691	0.742
	LR	0.831	0.728	0.711	0.741
	GNB	0.837	0.709	0.631	0.777
	KNN	0.741	0.627	0.633	0.624
cRP	SVM	0.802	0.691	0.589	0.776
	LR	0.811	0.710	0.651	0.756
	GNB	0.802	0.681	0.504	0.824
	KNN	0.692	0.635	0.524	0.723

Abbreviations: ACC, accuracy; AUC, area under the curve; CRP, c‐reactive protein; CrP, Clinical‐Pathomic model; CRp, Clinical‐Radiomic model; CRP, Clinical‐Radiomic‐Pathomic model; cRP, Radiomic‐Pathomic model; GNB, Gaussian Naive Bayes; KNN, K neighbor algorithm; LR, logistic regression; SVM, Support Vector Machine.

### 4.4 Prognostic performance of the comprehensive nomogram

**FIGURE 5:**
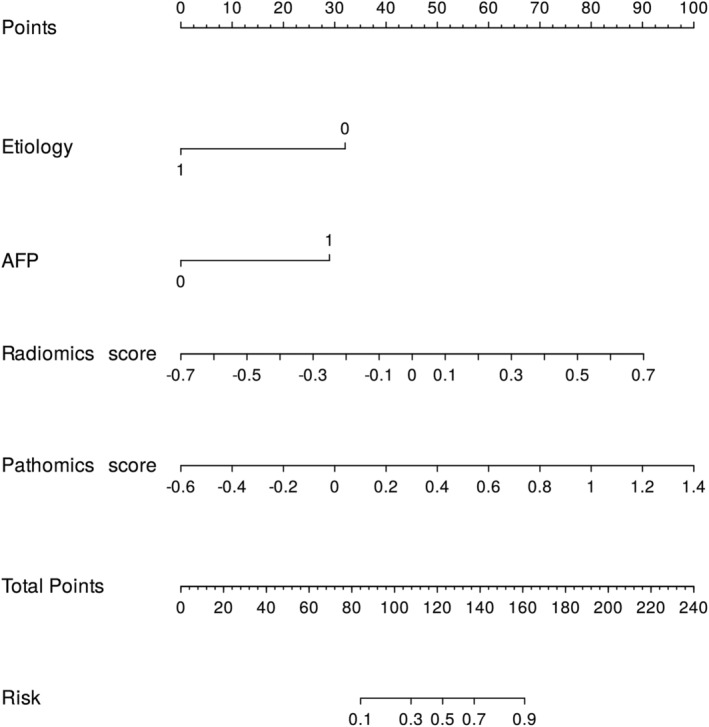
The nomogram of the multimodal model for identifying early recurrence. Etiology (0, HBV; 1, Other); AFP (0, ≤400;>400 ng/mL).

For ease of clinical application, based on the entire dataset, we constructed nomograms using the best clinical features, radiomics, and pathomics scores to represent the model results visually. Furthermore, the AUC and calibration curves of the composite nomogram indicated high accuracy and reliability of our model predictions, making it a valid tool for clinical practice in predicting the risk of early recurrence (Figure 5, Figure S2, and Table S5).

## 5 DISCUSSION

In this study, a multimodal model combining clinical radiomic pathomics features was constructed and validated by five‐fold cross‐validation to predict the risk of early recurrence of HCC after radical resection. The multimodal model features proved to have better performance than the bimodal model. In addition, the SVM constructed based on multimodal features outperformed other machine learning algorithms in predicting early recurrence. The machine learning model developed in this study provides essential support for more accurate personalized treatment plans and helps to optimize treatment decisions further, reduce the postoperative recurrence rate, and improve patients' quality of life.

In recent years, radiomics, as an emerging technology, can provide a large amount of information on morphological features, texture features, etc., which reflect the complexity of tumors and predict the growth and metastasis trends of tumors, and can be used to predict the risk of tumor recurrence.^35^, ^36^ However, there are still some limitations in using radiomics technology alone, such as inconsistency and uncertainty in feature extraction.^37^ To overcome these issues, researchers have combined radiomics technology with clinical features to obtain more comprehensive tumor information and improve predictive performance. Several recent studies have demonstrated that combining radiomics and clinical features can improve the accuracy of recurrence prediction in liver and pancreatic cancers and obtain better predictive performance than alone.^11^, ^12^, ^38^ Similarly, in our study, we found that both etiology and increased AFP can independently increase the risk of HCC recurrence, and when we combined radiomics features with clinical features, we found a significant improvement in predictive performance, consistent with previous research results.^39^, ^40^ It is worth noting that in our previous similar study, etiology, AFP, and tumor size were selected for clinical features, and there was a difference in the radiomic features selected for the two studies.^41^ This difference may be related to factors such as the number and composition of patients and the definition of recurrence.

With the continuous development of WSI technology, pathology‐based analysis of tissue pathology slides has been widely used in tumors.^42^, ^43^ In contrast to the traditional qualitative diagnosis of pathology, which relies on the subjective experience of pathologists, histopathology transforms pathological images into high‐fidelity, high‐throughput datasets through digital technology. This innovative approach enables quantitative analysis of pathology, resulting in a series of quantitative metrics, such as image quality features, image intensity features, image colocalization features, etc., which make pathological diagnosis more objective and reliable. Recent studies have shown that machine learning algorithms can accurately predict patients' survival and tumor grading by analyzing bladder cancer WSI.^44^ In addition, pathological proteomics has also demonstrated exemplary performance in predicting ovarian and liver cancer recurrence.^19^, ^45^ In our study, various machine learning algorithms were modeled and compared using clinical and pathological features, and a highly accurate and reliable clinical pathological proteomics model was ultimately developed, further validating its reliability in predicting liver cancer recurrence. It is important to note that the information provided by pathomics digs deep into the microscopic level of the tumor cells and their subcellular structures, which includes an exhaustive description of the nuclear morphology, chromatin distribution, organelle organization, and cytoplasmic structure, which is a direct reflection of the tumor's malignancy and therapeutic sensitivity. In contrast, the information presented by radiomics tends to reveal the macroscopic features of the tumor tissue. It captures the tumor's overall shape, size, location, and relative relationship with surrounding tissues through medical imaging techniques. This information emphasizes the general characteristics of the tumor tissue in terms of intensity distribution, density differences, and spatial relationships.^20^, ^21^ Therefore, combining radiomics and pathomics features can comprehensively capture both macroscopic and microstructural features of tumors and different dimensions of information at the tissue and cellular levels, which can help to understand the nature of the disease more accurately and guide therapeutic decisions.

Our study aims to explore predictive methods for early the recurrence of HCC. However, we found no reports on the joint prediction of clinical, radiomics, and pathomics features for the early recurrence of HCC. Based on 107 patients, two radiomics features, three pathomics features, and two clinical features were finally selected. We compare multiple learning algorithms and different types of features to determine the most favorable method for performance, ultimately developing a multimodal CRP‐SVM model with predictive ability. The main reasons for the model's excellent predictive ability are as follows: (a) data sources: clinical, radiomics, and pathomics features from different dimensions can be used to evaluate tumors and predict disease prognosis and treatment outcomes more effectively^46^; (b) data selection: the multi‐factor regression algorithm and LASSO algorithm can effectively reduce data overfitting, filter out a large number of features that have little impact on repetition, and improve prediction accuracy; (c) modeling method: SVM has the advantages of dealing with nonlinear problems in high‐dimensional space, processing small sample data, adapting to different types of datasets, avoiding local minimum value problems, and strong generalization ability^29^; (d) validation method: although our study is a small sample study, we implemented 5‐fold cross‐validation to balance interclass deviation.^47^

Finally, we constructed a more readable and usable visual nomogram based on the radiomics score, pathomics score, and clinical features to provide decision support for clinicians to provide personalized treatment. First, by analyzing each patient's comprehensive radiomic, pathomic, and clinical features, physicians can better understand the patient's probability of disease risk and develop a personalized treatment plan. Second, predictive assessment using this model can more accurately predict a patient's risk of early recurrence and develop personalized long‐term follow‐up plans and targeted monitoring. However, it is worth noting that this customized approach to treatment and prognostic assessment also faces some challenges. First, the approach may require additional technology and resources, including advanced imaging techniques and pathology analysis. Second, biological differences in individual patients may lead to some model limitations. Therefore, when implementing this approach, we need to weigh its potential benefits against cost, feasibility, and applicability.

Although we have identified some promising findings, our study has some limitations. First, our dataset was retrospectively collected from a single institution with limited patient numbers. Although we implemented 5‐fold cross‐validation in our study to mitigate this limitation, future research should use larger, multi‐center prospective datasets. Second, many factors can affect the reproducibility of features, such as segmentation methods and radiomics feature extraction software. In this study, we took measures such as having two radiologists consistently delineate the CT image's ROI to improve the reproducibility of features. However, these measures can only partially eliminate the problem of reproducibility. Third, pathomics features were sourced from tissue biopsies, which may have tissue heterogeneity and sampling bias.

## 6 CONCLUSION

A multimodal model, which combines clinical, radiomics, and pathomics features, was developed to predict early recurrence in HCC patients who underwent radical surgery, demonstrating high predictive performance. This model could provide valuable references for personalized treatment and monitoring plans for HCC patients.

## AUTHOR CONTRIBUTIONS

**Qu Xie:** Conceptualization (equal); data curation (equal); formal analysis (equal); validation (equal); visualization (equal); writing – original draft (equal). **Zeyin Zhao:** Conceptualization (equal); software (equal). **Yanzhen Yang:** Formal analysis (equal); methodology (equal). **Xiaohong Wang:** Methodology (equal); project administration (equal). **Wei Wu:** Investigation (equal). **Haitao Jiang:** Conceptualization (equal); investigation (equal). **Weiyuan Hao:** Project administration (equal). **Ruizi Peng:** Conceptualization (equal); project administration (equal). **Cong Luo:** Conceptualization (equal); data curation (equal); formal analysis (equal); funding acquisition (equal).

## CONFLICT OF INTEREST STATEMENT

The authors declare that the research was conducted without any commercial or financial relationships that could be construed as a potential conflict of interest.

## ETHICS STATEMENT

Patients were recruited following the Helsinki Declaration. The Ethics Committee of Zhejiang Cancer Hospital approved this retrospective study, and the requirement for informed patient consent was waived (IRB‐2022‐503).

## Supporting information


Figure S1.

Figure S2.

Table S1.

Table S2.

Table S3.

Table S4.

Table S5.

## Data Availability

The datasets analyzed during the current study are available from the corresponding author on reasonable request.
