# Supplementary figures and images for: A clinical‐radiomic‐pathomic model for prognosis prediction in patients with hepatocellular carcinoma after radical resection
Source: Cancer Med. 2024 Jun 12;13(11):e7374. doi: 10.1002/cam4.7374 (PMC11167608; doi:10.1002/cam4.7374)

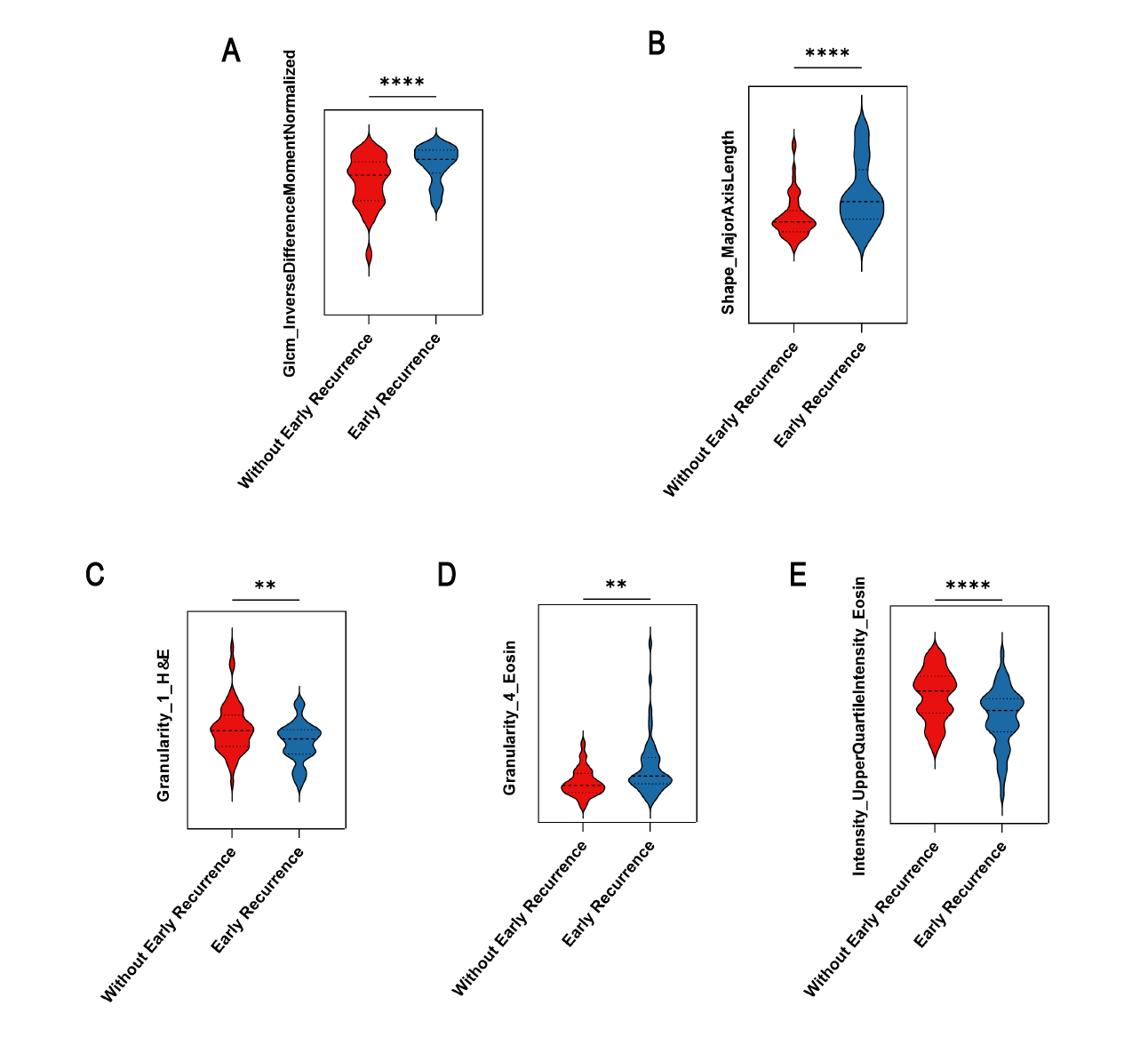

Supplement: Supplementary file 1 — Figure S1. [file CAM4-13-e7374-s004.tif]

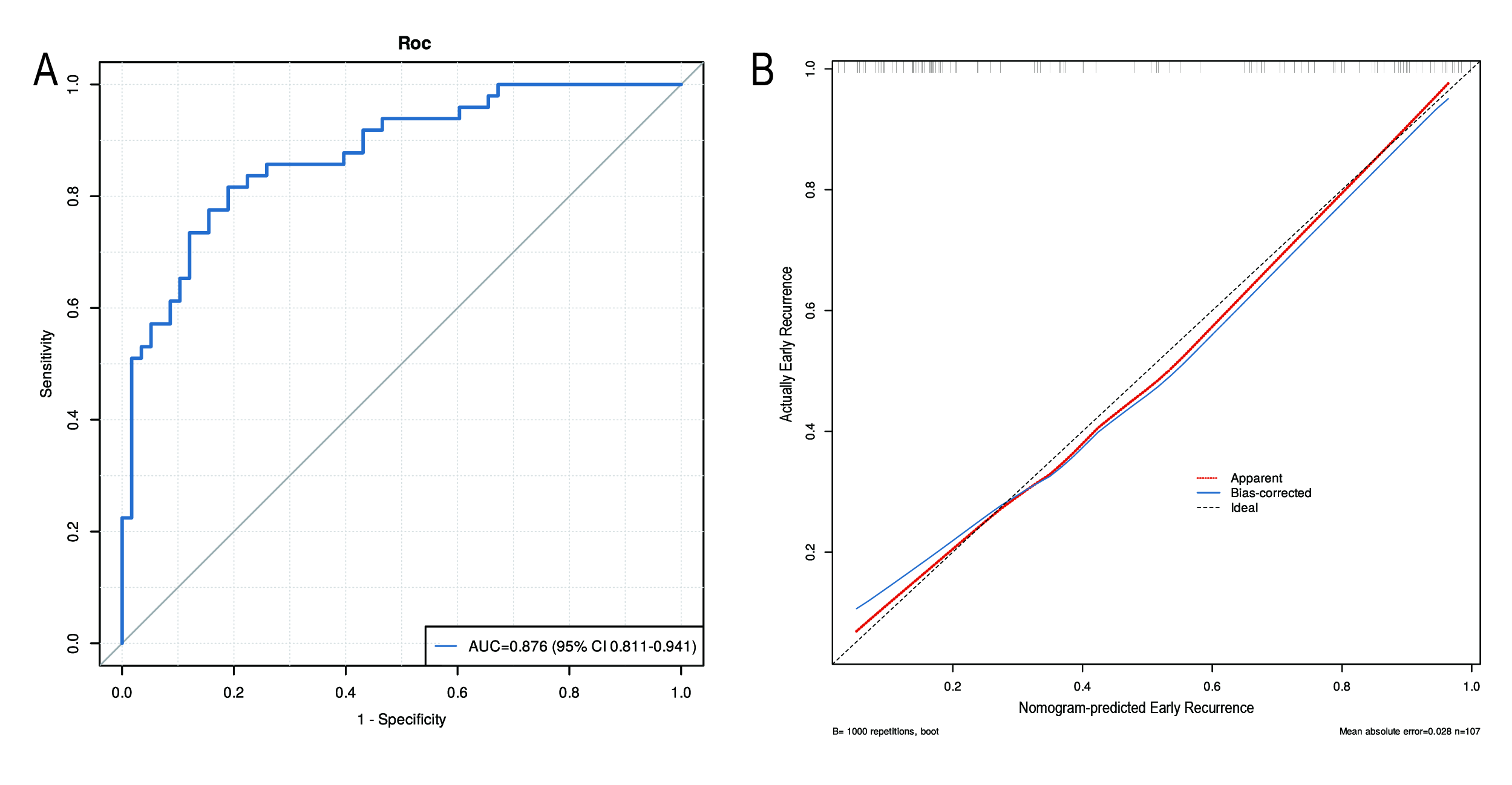

Supplement: Supplementary file 2 — Figure S2. [file CAM4-13-e7374-s002.tif]
