# Supplementary material for: A clinical‐radiomic‐pathomic model for prognosis prediction in patients with hepatocellular carcinoma after radical resection
Source: Cancer Med. 2024 Jun 12;13(11):e7374. doi: 10.1002/cam4.7374 (PMC11167608; doi:10.1002/cam4.7374)
Supplement: Supplementary file 3 — Table S1. [file CAM4-13-e7374-s001.docx]

**Table S1. Baseline characteristics of overall recurrence.**

| **Characteristic** | | **Non-overall recurrence(n=39)** | **Overall-recurrence(n=68)** | **Univariable Logistic Regression** | | **Multivariable Logistic Regression** | |
| --- | --- | --- | --- | --- | --- | --- | --- |
|  |  |  |  | **OR (95%CI)** | ***P*** | **OR (95%CI)** | ***P*** |
| Patient demographics | |  |  |  |  |  |  |
| Age (mean (SD)) | 59.36(9.09) | 57.07(11.05) | 0.979(0.941,1.018) | 0.278 |  |  |  |
| Sex (female/ male) | 4 (10.3)/35 (89.7) | 10 (14.7)/58 (85.3) | 0.663(0.193,2.275) | 0.513 |  |  |  |
| Etiology (HBV/Other) | 26 (66.7)/13 (33.3) | 60 (88.2)/8 (11.8) | 0.267(0.099,0.720) | **0.009** | 0.222(0.053,0.934) | **0.040** |  |
| Liver cirrhosis (NO/Yes) | 15 (38.5)/24 (61.5) | 32 (47.1)/36 (52.9) | 0.703(0.315,1.568) | 0.389 |  |  |  |
| BCLC staging (0orA/B) | 37 (94.9)/2 (5.1) | 54 (79.4)/14 (20.6) | 4.796(1.029,22.362) | **0.046** | 4.314(0.721,25.830) | 0.109 |  |
| Adjuvant therapy (No/Yes) | 26 (66.7)/13 (33.3) | 36 (52.9)/32 (47.1) | 1.778(0.784,4.031) | 0.168 |  |  |  |
| Laboratory parameters | |  |  |  |  |  |  |
| TP(≤65/＞65 g/L) | 6 (15.4)/33 (84.6) | 10 (14.7)/58 (85.3) | 1.055(0.352,3.164) | 0.925 |  |  |  |
| ALB(≤40/＞40 g/L) | 9 (23.1)/30 (76.9) | 25 (36.8)/43 (63.2) | 0.516(0.211,1.261) | 0.147 |  |  |  |
| A/G(≤1.5/＞1.5 g/L) | 5 (12.8)/34 (87.2) | 12 (17.6)/56 (82.4) | 0.686(0.222,2.118) | 0.513 |  |  |  |
| ALT(≤50/＞50 U/L) | 28 (71.8)/11 (28.2) | 52 (76.5)/16 (23.5) | 0.783(0.320,1.916) | 0.592 |  |  |  |
| AST(≤40/＞40 U/L) | 25 (64.1)/14 (35.9) | 41 (60.3)/27 (39.7) | 1.176(0.521,2.656) | 0.697 |  |  |  |
| ALP(≤45/＞45 U/L) | 35 (89.7)/4 (10.3) | 61 (89.7)/7 (10.3) | 1.004(0.275,3.673) | 0.995 |  |  |  |
| GGT(≤60/＞60 U/L) | 24 (61.5)/15 (38.5) | 33 (48.5)/35 (51.5) | 1.697(0.761,3.782) | 0.196 |  |  |  |
| TBIL(≤20/＞20 µmol/L) | 33 (84.6)/6 (15.4) | 63 (92.6)/5 (7.4) | 0.437(0.124,1.538) | 0.197 |  |  |  |
| DBIL(≤7/＞7 µmol/L) | 33 (84.6)/6 (15.4) | 64 (94.1)/4 (5.9) | 0.344(0.091,1.304) | 0.116 |  |  |  |
| CRP (≤10/＞10 mg/L) | 33 (84.6)/6 (15.4) | 58 (85.3)/10 (14.7) | 0.948(0.316,2.845) | 0.925 |  |  |  |
| WBC(≤3.5/＞3.5 x10^9^/L) | 2 (5.1)/37 (94.9) | 3 (4.4)/65 (95.6) | 1.171(0.187,7.331) | 0.866 |  |  |  |
| Neut (≤1.8/＞1.8 x10^9^/L ) | 1 (2.6)/38 (97.4) | 4 (5.9)/64 (94.1) | 0.421(0.045,3.907) | 0.447 |  |  |  |
| Lymp (≤1.1/＞1.1 x10^9^/L) | 8 (20.5)/31 (79.5) | 7 (10.3)/61 (89.7) | 2.249(0.747,6.774) | 0.150 |  |  |  |
| NLR(≤3/＞3) | 31 (79.5)/8 (20.5) | 50 (73.5)/18 (26.5) | 1.395(0.542,3.592) | 0.490 |  |  |  |
| Plt(≤125/＞125 x10^9^/L ) | 8 (20.5)/31 (79.5) | 12 (17.6)/56 (82.4) | 1.204(0.445,3.262) | 0.715 |  |  |  |
| PLR (≤125＞/125) | 27 (69.2)/12 (30.8) | 57 (83.8)/11 (16.2) | 0.434(0.170,1.109) | **0.081** | 0.849(0.217,3.313) | 0.813 |  |
| INR (≤1/＞1) | 38 (97.4)/1 (2.6) | 66 (97.1)/2 (2.9) | 1.152(0.101,13.125) | 0.910 |  |  |  |
| PT (≤14/＞14 seconds) | 35 (89.7)/4 (10.3) | 66 (97.1)/2 (2.9) | 0.265(0.046,1.520) | 0.136 |  |  |  |
| AFP(≤400/＞400 ng/ml) | 20 (51.3)/19 (48.7) | 19 (27.9)/49 (72.1) | 2.715(1.194,6.173) | **0.017** | 2.414(0.959,6.078) | 0.061 |  |
| CEA(≤5/＞5 ng/ml) | 36 (92.3)/3 (7.7) | 61 (89.7)/7 (10.3) | 1.377(0.335,5.662) | 0.657 |  |  |  |
| Child-Pugh Class(A/B) | 33 (84.6)/6 (15.4) | 45 (66.2)/23 (33.8) | 2.811(1.029,7.676) | **0.044** | 2.281(0.765,6.797) | 0.139 |  |
| Surgical pathology information | |  |  |  |  |  |  |
| Tumor number(≤3/＞3) | 38 (97.4)/1 (2.6) | 60 (88.2)/8 (11.8) | 5.067(0.609,42.133) | 0.133 |  |  |  |
| Tumor size(≤5/＞5 cm) | 29 (74.4)/10 (25.6) | 35 (51.5)/33 (48.5) | 2.734(1.155,6.473) | **0.022** | 2.008(0.750,5.375) | 0.165 |  |
| Capsular invasion (No/Yes) | 29 (74.4)/10 (25.6) | 40 (58.8)/28 (41.2) | 2.030(0.854,4.826) | 0.109 |  |  |  |
| Neural invasion (No/Yes) | 33 (84.6)/6 (15.4) | 48 (70.6)/20 (29.4) | 2.292(0.831,6.319) | 0.109 |  |  |  |
