# Supplementary material for: A clinical‐radiomic‐pathomic model for prognosis prediction in patients with hepatocellular carcinoma after radical resection
Source: Cancer Med. 2024 Jun 12;13(11):e7374. doi: 10.1002/cam4.7374 (PMC11167608; doi:10.1002/cam4.7374)
Supplement: Supplementary file 4 — Table S2. [file CAM4-13-e7374-s006.docx]

**Table S2. Radiomics features.**

| **Feature classification(N)^a^** | **Feature description** |  | **Feature classification(N)^a^** | **Feature description** |
| --- | --- | --- | --- | --- |
| **GLCM ^b^ Features**  **(N=24)** | glcm_Autocorrelation |  | **GLCM ^b^ Features**  **(N=24)** | glcm_MaximumProbability |
|  | glcm_JointAverage |  |  | glcm_SumAverage |
|  | glcm_ClusterProminence |  |  | glcm_SumEntropy |
|  | glcm_ClusterShade |  |  | glcm_SumofSquares |
|  | glcm_ClusterTendency |  | **GLSZM^b^ Features**  **(N=16)** | glszm_SmallAreaEmphasis |
|  | glcm_Contrast |  |  | glszm_LargeAreaEmphasis |
|  | glcm_Correlation |  |  | glszm_GrayLevelNonUniformity |
|  | glcm_DifferenceAverage |  |  | glszm_GrayLevelNonUniformityNormalized |
|  | glcm_DifferenceEntropy |  |  | glszm_SizeZoneNonUniformity |
|  | glcm_DifferenceVariance |  |  | glszm_SizeZoneNonUniformityNormalized |
|  | glcm_JointEnergy |  |  | glszm_ZonePercentage |
|  | glcm_JointEntropy |  |  | glszm_GrayLevelVariance |
|  | glcm_InformationalMeasureofCorrelation1 |  |  | glszm_ZoneVariance |
|  | glcm_InformationalMeasureofCorrelation2 |  |  | glszm_ ZoneEntropy |
|  | glcm_InverseDifferenceMoment |  |  | glszm_ LowGrayLevelZoneEmphasis |
|  | glcm_MaximalCorrelationCoefficient |  |  | glszm_ HighGrayLevelZoneEmphasis |
|  | glcm_InverseDifferenceMomentNormalized |  |  | glszm_ SmallAreaLowGrayLevelEmphasis |
|  | glcm_InverseDifference |  |  | glszm_ SmallAreaHighGrayLevelEmphasis |
|  | glcm_InverseDifferenceNormalized |  |  | glszm_ LargeAreaLowGrayLevelEmphasis |
|  | glcm_InverseVariance |  |  | glszm_ LargeAreaHighGrayLevelEmphasis |

| **Feature classification(N)^a^** | **Feature description** |  | **Feature classification(N)^a^** | **Feature description** |
| --- | --- | --- | --- | --- |
| **NGTDM^b^ Features**  **(N=5)** | ngtdm_Coarseness |  | **First Order Features**  **(N=18)** | firstorder**_**Energy |
|  | ngtdm_Busyness |  |  | firstorder**_**TotalEnergy |
|  | ngtdm_Complexity |  |  | firstorder**_**Entropy |
|  | ngtdm_Strength |  |  | firstorder**_**Minimum |
|  | ngtdm_Contrast |  |  | firstorder**_**10thpercentile |
| **Shape Features**  **(N=14)** | shape_MeshVolume |  |  | firstorder**_**90thpercentile |
|  | shape_VoxelVolume |  |  | firstorder**_**Maximum |
|  | shape_SurfaceArea |  |  | firstorder**_**Mean |
|  | shape_SurfaceAreatoVolumeratio |  |  | firstorder**_**Median |
|  | shape_Sphericity |  |  | firstorder**_**InterquartileRange |
|  | shape_Maximum3Ddiameter |  |  | firstorder**_**Range |
|  | shape_Maximum2Ddiameter(Slice) |  |  | firstorder**_**MeanAbsoluteDeviation |
|  | shape_Maximum2Ddiameter(Column) |  |  | firstorder**_**RobustMeanAbsoluteDeviation |
|  | shape_Maximum2Ddiameter(Row) |  |  | firstorder**_**RootMeanSquared |
|  | shape_MajorAxisLength |  |  | firstorder**_**Skewness |
|  | shape_MinorAxisLength |  |  | firstorder**_**Kurtosis |
|  | shape_LeastAxisLength |  |  | firstorder**_**Variance |
|  | shape_Elongation |  |  | firstorder**_**Uniformity |
|  | shape_Flatness |  |  |  |

| **Feature classification(N)^a^** | **Feature description** |  | **Feature classification(N)^a^** | **Feature description** | |
| --- | --- | --- | --- | --- | --- |
| **GLRLM^b^ Features**  **(N=16)** | glrlm_ShortRunEmphasis |  | **GLDM^b^ Features**  **(N=14)** | gldm_SmallDependenceEmphasis | |
|  | glrlm_LongRunEmphasis |  |  | gldm_LargeDependenceEmphasis | |
|  | glrlm_GrayLevelNonUniformity |  |  | gldm_GrayLevelNonUniformity | |
|  | glrlm_GrayLevelNonUniformityNormalized |  |  | gldm_DependenceNonUniformity | |
|  | glrlm_RunLengthNonUniformity |  |  | gldm_DependenceNonUniformityNormalized | |
|  | glrlm_RunLengthNonUniformityNormalized |  |  | gldm_GrayLevelVariance | |
|  | glrlm_RunPercentage |  |  | gldm_DependenceVariance | |
|  | glrlm_GrayLevelVariance |  |  | gldm_DependenceEntropy | |
|  | glrlm_RunVariance |  |  | gldm_LowGrayLevelEmphasis | |
|  | glrlm_RunEntropy |  |  | gldm_HighGrayLevelEmphasis | |
|  | glrlm_ LowGrayLevelRunEmphasis |  |  | gldm_SmallDependenceLowGrayLevelEmphasis | |
|  | glrlm_ HighGrayLevelRunEmphasis |  |  | gldm_SmallDependenceHighGrayLevelEmphasis | |
|  | glrlm_ ShortRunLowGrayLevelEmphasis |  |  | gldm_LargeDependenceLowGrayLevelEmphasis | |
|  | glrlm_ ShortRunHighGrayLevelEmphasis |  |  | gldm_LargeDependenceHighGrayLevelEmphasis | |
|  | glrlm_ LongRunLowGrayLevelEmphasis |  |  |  | |
|  | glrlm_ LongRunHighGrayLevelEmphasis |  |  |  |  |

**Note: ^a^(N) indicated the total number of features in distinct group.**

**^b^GLCM, Gray Level Co-occurrence Matrix; GLSZM, Gray-level size zone matrix; GLRLM, Gray Level Run Length Matrix; NGTDM, Neighbouring Gray Tone Difference Matrix;** **GLDM,** **Gray Level Dependence Matrix.**

***The abbreviated representation of feature types**
