# Supplementary material for: A clinical‐radiomic‐pathomic model for prognosis prediction in patients with hepatocellular carcinoma after radical resection
Source: Cancer Med. 2024 Jun 12;13(11):e7374. doi: 10.1002/cam4.7374 (PMC11167608; doi:10.1002/cam4.7374)
Supplement: Supplementary file 5 — Table S3. [file CAM4-13-e7374-s007.docx]

**Table S3. Pathomics features.**

| **Feature classification** | **Feature description** |
| --- | --- |
| Image quality features | FocusScore_α |
|  | LocalFocusScore_α |
|  | Correlation_α |
|  | PowerLogLogSlope_α |
| Image intensity features | MeanIntensity_α |
|  | MedianIntensity_α |
|  | StdIntensity_α |
|  | MADIntensity_α |
|  | LowerQuartileIntensity_α |
|  | UpperQuartileIntensity_α |
|  | Threshold_α |
| Image colocalization features | Correlation_Eosin_Hematoxylin |
|  | Correlation_Costes_Eosin_Hematoxylin |
|  | Correlation_Costes_Hematoxylin_Eosin |
|  | Correlation_Manders_Eosin_Hematoxylin |
|  | Correlation_Manders_Hematoxylin_Eosin |
|  | Correlation_Overlap_Eosin_Hematoxylin |
|  | Correlation_RWC_Eosin_Hematoxylin |
|  | Correlation_RWC_Hematoxylin_Eosin |
|  | Correlation_Slope_Eosin_Hematoxylin |
| Image granularity features | Granularity_β_α |

**Notes:** The symbol α corresponds to the image type, which encompasses hematoxylin, eosin, and H&E.

The symbol β stands for the granular spectrum, which spans from 1 to 16.

H&E, hematoxylin and eosin
