# Supplementary material for: A clinical‐radiomic‐pathomic model for prognosis prediction in patients with hepatocellular carcinoma after radical resection
Source: Cancer Med. 2024 Jun 12;13(11):e7374. doi: 10.1002/cam4.7374 (PMC11167608; doi:10.1002/cam4.7374)
Supplement: Supplementary file 6 — Table S4. [file CAM4-13-e7374-s005.docx]

**Table S4. Variations in the predictive performance of four machine learning algorithms modeled across four types of datasets employing five-fold cross-validations.**

| **Models** | **Classifiers** | **Fold** | **AUC** | **ACC** | **Sensitivity** | **Specificity** |
| --- | --- | --- | --- | --- | --- | --- |
| CRP | SVM | Fold 1 | 0.825 | 0.818 | 0.800 | 0.833 |
|  |  | Fold 2 | 0.883 | 0.818 | 0.700 | 0.917 |
|  |  | Fold 3 | 0.731 | 0.714 | 0.556 | 0.833 |
|  |  | Fold 4 | 0.982 | 0.810 | 1.000 | 0.636 |
|  |  | Fold 5 | 0.891 | 0.762 | 0.600 | 0.909 |
|  | LR | Fold 1 | 0.833 | 0.818 | 0.800 | 0.833 |
|  |  | Fold 2 | 0.883 | 0.773 | 0.700 | 0.833 |
|  |  | Fold 3 | 0.704 | 0.667 | 0.556 | 0.750 |
|  |  | Fold 4 | 0.982 | 0.810 | 1.000 | 0.636 |
|  |  | Fold 5 | 0.800 | 0.762 | 0.600 | 0.909 |
|  | GNB | Fold 1 | 0.842 | 0.818 | 0.700 | 0.917 |
|  |  | Fold 2 | 0.875 | 0.864 | 0.700 | 1.000 |
|  |  | Fold 3 | 0.750 | 0.714 | 0.556 | 0.833 |
|  |  | Fold 4 | 0.955 | 0.810 | 1.000 | 0.636 |
|  |  | Fold 5 | 0.873 | 0.667 | 0.500 | 0.818 |
|  | KNN | Fold 1 | 0.675 | 0.682 | 0.700 | 0.667 |
|  |  | Fold 2 | 0.692 | 0.636 | 0.600 | 0.667 |
|  |  | Fold 3 | 0.727 | 0.714 | 0.556 | 0.833 |
|  |  | Fold 4 | 0.900 | 0.857 | 1.000 | 0.727 |
|  |  | Fold 5 | 0.723 | 0.667 | 0.400 | 0.909 |
| CRp | SVM | Fold 1 | 0.883 | 0.818 | 0.800 | 0.833 |
|  |  | Fold 2 | 0.883 | 0.773 | 0.700 | 0.833 |
|  |  | Fold 3 | 0.676 | 0.667 | 0.556 | 0.750 |
|  |  | Fold 4 | 0.909 | 0.810 | 1.000 | 0.636 |
|  |  | Fold 5 | 0.764 | 0.714 | 0.600 | 0.818 |
|  | LR | Fold 1 | 0.850 | 0.727 | 0.900 | 0.583 |
|  |  | Fold 2 | 0.825 | 0.727 | 0.600 | 0.833 |
|  |  | Fold 3 | 0.741 | 0.619 | 0.556 | 0.667 |
|  |  | Fold 4 | 0.909 | 0.714 | 1.000 | 0.455 |
|  |  | Fold 5 | 0.727 | 0.762 | 0.700 | 0.818 |
|  | GNB | Fold 1 | 0.842 | 0.727 | 0.800 | 0.667 |
|  |  | Fold 2 | 0.817 | 0.727 | 0.600 | 0.833 |
|  |  | Fold 3 | 0.741 | 0.619 | 0.444 | 0.750 |
|  |  | Fold 4 | 0.855 | 0.667 | 0.900 | 0.455 |
|  |  | Fold 5 | 0.782 | 0.714 | 0.600 | 0.818 |
|  | KNN | Fold 1 | 0.738 | 0.682 | 0.600 | 0.750 |
|  |  | Fold 2 | 0.775 | 0.773 | 0.700 | 0.833 |
|  |  | Fold 3 | 0.579 | 0.619 | 0.556 | 0.667 |
|  |  | Fold 4 | 0.855 | 0.810 | 1.000 | 0.636 |
|  |  | Fold 5 | 0.700 | 0.714 | 0.600 | 0.818 |
| CrP | SVM | Fold 1 | 0.825 | 0.818 | 0.800 | 0.833 |
|  |  | Fold 2 | 0.917 | 0.727 | 0.800 | 0.667 |
|  |  | Fold 3 | 0.611 | 0.619 | 0.556 | 0.667 |
|  |  | Fold 4 | 0.918 | 0.810 | 1.000 | 0.636 |
|  |  | Fold 5 | 0.864 | 0.619 | 0.300 | 0.909 |
|  | LR | Fold 1 | 0.800 | 0.727 | 0.800 | 0.667 |
|  |  | Fold 2 | 0.933 | 0.864 | 0.800 | 0.917 |
|  |  | Fold 3 | 0.611 | 0.619 | 0.556 | 0.667 |
|  |  | Fold 4 | 0.936 | 0.762 | 1.000 | 0.545 |
|  |  | Fold 5 | 0.873 | 0.667 | 0.400 | 0.909 |
|  | GNB | Fold 1 | 0.808 | 0.727 | 0.700 | 0.750 |
|  |  | Fold 2 | 0.900 | 0.818 | 0.800 | 0.833 |
|  |  | Fold 3 | 0.630 | 0.619 | 0.556 | 0.667 |
|  |  | Fold 4 | 0.891 | 0.762 | 0.900 | 0.636 |
|  |  | Fold 5 | 0.955 | 0.619 | 0.200 | 1.000 |
|  | KNN | Fold 1 | 0.588 | 0.500 | 0.500 | 0.500 |
|  |  | Fold 2 | 0.846 | 0.636 | 0.800 | 0.500 |
|  |  | Fold 3 | 0.648 | 0.667 | 0.667 | 0.667 |
|  |  | Fold 4 | 0.855 | 0.714 | 0.900 | 0.545 |
|  |  | Fold 5 | 0.768 | 0.619 | 0.300 | 0.909 |
| cRP | SVM | Fold 1 | 0.733 | 0.636 | 0.500 | 0.750 |
|  |  | Fold 2 | 0.883 | 0.773 | 0.700 | 0.833 |
|  |  | Fold 3 | 0.685 | 0.619 | 0.444 | 0.750 |
|  |  | Fold 4 | 0.955 | 0.762 | 0.900 | 0.636 |
|  |  | Fold 5 | 0.755 | 0.667 | 0.400 | 0.909 |
|  | LR | Fold 1 | 0.783 | 0.682 | 0.600 | 0.750 |
|  |  | Fold 2 | 0.875 | 0.773 | 0.700 | 0.833 |
|  |  | Fold 3 | 0.713 | 0.714 | 0.556 | 0.833 |
|  |  | Fold 4 | 0.964 | 0.762 | 1.000 | 0.545 |
|  |  | Fold 5 | 0.718 | 0.619 | 0.400 | 0.818 |
|  | GNB | Fold 1 | 0.825 | 0.727 | 0.500 | 0.917 |
|  |  | Fold 2 | 0.792 | 0.727 | 0.600 | 0.833 |
|  |  | Fold 3 | 0.731 | 0.619 | 0.222 | 0.917 |
|  |  | Fold 4 | 0.945 | 0.762 | 0.900 | 0.636 |
|  |  | Fold 5 | 0.718 | 0.571 | 0.300 | 0.818 |
|  | KNN | Fold 1 | 0.650 | 0.682 | 0.600 | 0.750 |
|  |  | Fold 2 | 0.675 | 0.682 | 0.500 | 0.833 |
|  |  | Fold 3 | 0.611 | 0.476 | 0.222 | 0.667 |
|  |  | Fold 4 | 0.823 | 0.714 | 0.900 | 0.545 |
|  |  | Fold 5 | 0.700 | 0.619 | 0.400 | 0.818 |
