# Supplementary material for: A clinical‐radiomic‐pathomic model for prognosis prediction in patients with hepatocellular carcinoma after radical resection
Source: Cancer Med. 2024 Jun 12;13(11):e7374. doi: 10.1002/cam4.7374 (PMC11167608; doi:10.1002/cam4.7374)
Supplement: Supplementary file 7 — Table S5. [file CAM4-13-e7374-s003.docx]

| **Predictor** | **Estimate (SE)** | ***p*** | **Odds Ratio** | **Lower** | **Upper** |
| --- | --- | --- | --- | --- | --- |
| (Intercept) | -0.879(0.462) | 0.057 | 0.415 | 0.157 | 0.987 |
| Etiology | -1.835(0.737) | 0.013 | 0.160 | 0.032 | 0.607 |
| AFP | 1.659(0.556) | 0.003 | 5.254 | 1.837 | 16.637 |
| Radiomics score | 3.691(1.143) | 0.001 | 40.067 | 4.977 | 462.126 |
| Pathomics score | 2.864(0.933) | 0.002 | 17.526 | 3.207 | 127.985 |

**Table S5. The nomogram analysis of early recurrence.**
